# Childbed Nursing

**Published:** 1902-07

**Authors:** 


Childbed Nursing.—A manual of childbed nursing, with notes on infant feeding. By Charles Jewett, A. M., M. D., Sc. D., professor of obstetrics and diseases of women in the Long Island College Hospital. Fifth edition, revised and enlarged. New York: E. B. Treat & Co., 241-243 West Twenty-third street; 1902. Cloth, 85 pages, $0.80 net.

The subjects treated in this little brochure are pregnancy, labor, puerperal period, care of the mother, prevention of childbed infection, care of the child, and artificial feeding. The book is but little more than a carefully prepared set of lecture notes, and is evidently intended for only nurses and mothers. The fact that it is from the pen of Dr. Jewett is a sufficient guarantee that it is well written.

